# Dwell Times Reveal Effects of Abstract Event Type on Attention Allocation

**DOI:** 10.1111/cogs.70169

**Published:** 2026-01-19

**Authors:** Jamie Yuen, Sarah Hye‐yeon Lee, Anna Papafragou

**Affiliations:** ^1^ Department of Linguistics University of Pennsylvania

**Keywords:** Event cognition, Boundedness, Attention, Dwell time, Aspect

## Abstract

The human mind can segment continuous streams of activity in the world into meaningful, discrete units known as events. However, not all events are created equal. We draw a distinction between *bounded* events (e.g., folding a handkerchief) that have a predictable structure that develops in distinct stages (i.e., a beginning, middle, and end) and a well‐defined endpoint, and *unbounded* events (e.g., waving a handkerchief) that lack such a well‐defined structure and endpoint. We predict that event boundedness affects attention allocation patterns over the course of the event. Here, we tested this prediction using a dwell time paradigm by measuring the time participants spent on each still frame of an activity. We found that event endpoints attracted increased attention compared to midpoints; importantly, this increase was significantly greater when people viewed bounded events compared to unbounded events. In addition, event endpoints attracted increased attention compared to event beginnings, but this pattern also interacted with event boundedness (Experiment 1). These results replicated even when a linguistic preview of the events was introduced (Experiment 2). We conclude that abstract internal event structure (specifically, event boundedness) affects attention allocation during online event apprehension.

## 1 Introduction

Humans are surrounded by rich, dynamic, and continuous input, yet are able to chunk this input into discrete *events*. This ability has been studied by having observers press a key to mark boundaries, or breakpoints, between events in a video of naturalistic human or human‐like action (Newtson, 1973; Newtson & Engquist, 1976). Evidence suggests that there is robust agreement on event boundaries across individuals (e.g., Newtson, 1973, Zacks, Tversky, & Iyer, 2001), and event segmentation appears to occur spontaneously as people process sequences of ongoing activity (Saylor & Baldwin, 2004; Huff, Papenmeier, & Zacks, 2012; Zacks et al., 2001).

### 1.1 Dwell times as a window onto event structure

Previous studies have used the dwell time paradigm to probe mechanisms of event segmentation and representation (Hard, Recchia, & Tversky, 2019, 2011; Kosie & Baldwin, 2019a, 2019b, 2021). In a dwell time experiment, activity sequences are converted into image slideshows, and observers advance through them at their own pace. Their dwell times—time spent looking (“dwelling”) at each slide—provide a measure of attention, with greater time spent looking at a slide indicating greater attention. In a pioneering study, Hard et al. (2011) presented to participants four familiar, potentially hierarchically organized activities (e.g., cleaning a dorm room, eating breakfast) in the form of slideshows and measured dwell times at each slide as participants proceeded through them at their own pace. Then, participants viewed the same activity in video form and segmented it into smaller events by marking where one event ended and another began (i.e., the event boundary). Dwell times were analyzed on slides that each participant later identified as being within‐event or at an event boundary. Increased dwell times were found at event boundary slides compared to within‐event slides, indicating that event boundaries elicit greater attention. Such enhanced attention to event boundaries is known as the “boundary advantage.” The boundary advantage has been found across different event exemplars (Sage & Baldwin, 2014), and even in preschoolers (Kosie & Baldwin, 2021), and appears to be linked to enhanced subsequent memory for event units (Gold, Zacks, & Flores, 2017; McGatlin, Newberry, & Bailey, 2018; see Yousif, Lee, Sherman, & Papafragou, 2024 for related findings with larger‐scale stimuli from outside the lab). More broadly, a variety of existing studies suggests that event boundaries—especially event endpoints—are salient within the representation of an event (Huff et al., 2012; Ongchoco & Scholl, 2019; Pettijohn & Radvansky, 2016; Schwan & Garsoffky, 2004; Swallow, Zacks, & Abrams, 2009; Zacks, Speer, Swallow, Braver, & Reynolds, 2007; Zacks & Tversky, 2001).

The finding that event boundaries yield greater attention aligns with Event Segmentation Theory (EST) (Zacks et al., 2007). EST proposes that people are constantly predicting what will happen next and, when happenings become increasingly unpredictable, and prediction errors emerge, an event boundary occurs, indicating where one event ends, and another begins (Zacks et al., 2007). According to EST, unpredictable, critical information is being relayed at event boundaries and warrants increased attention. Consistent with this idea, dwell times tend to increase just before, rather than in response to, the completion of an action (Hard et al., 2011). As viewers anticipate that an action is about to be completed, they increasingly direct more attention to the action. With increased attention, viewers can better process information at the boundary region (Hard, Meyer, & Baldwin, 2019).

### 1.2 Boundedness: Beyond a single notion of “event structure” and “event boundary”

The literature on event segmentation and processing reviewed earlier has largely treated all events as similar entities characterized by the same attentional signatures (including the “boundary advantage”). However, recent evidence shows that there are, in fact, foundational ontological differences in conceptual event types captured by the notion of *boundedness* (see Ji & Papafragou, 2020a, 2020b). *Bounded* events have a nonhomogeneous structure with distinct stages that lead to a well‐defined endpoint (or culmination). A girl folding a handkerchief is an example of a bounded event: its endpoint is defined as the point at which the handkerchief is entirely folded. By contrast, *unbounded* events have a homogenous structure that lacks a well‐defined endpoint and, therefore, may terminate at any arbitrary moment (Ji & Papafragou, 2020a, 2020b). A girl waving a handkerchief is an example of an unbounded event: this event could be considered to reach an end at any arbitrary time point when the waving action stops. The bounded‐unbounded distinction in the event domain is akin to the object‐substance distinction in the object domain: bounded events resemble objects (e.g., a book) because they possess well‐defined boundaries, and behave like canonical individuals; unbounded events, on the other hand, resemble substances (e.g., paper) because they lack well‐defined boundaries and are not canonical individuals (they cannot be individuated or counted; see Papafragou & Ji, 2023; Lee, Ji, & Papafragou, 2024, for empirical evidence of the event‐object parallel; cf. also Kuhn et al., 2021; Wellwood, Hespos, & Rips, 2018).

Within event cognition, boundedness should be understood as a perspective on events imposed by the human mind and not an objective feature of the world (Vurgun, Ji & Papafragou, 2024; Wagner & Carey, 2003). Boundedness is reflected in human language (via the expression of *telicity*; Filip, 1993; Folli & Harley, 2006; Krifka, 1992, 1998; van Hout, 2016). Most relevantly for present purposes, viewers have been found to be sensitive to boundedness: for instance, both adults and young children can classify novel visual events as being either bounded or unbounded (Ji & Papafragou, 2020a; 2020b). Furthermore, viewers process these two types of events differently in event cognition—for instance, by treating structural disruptions of the content of events as more problematic if the event is bounded than unbounded (Ji & Papafragou, 2020b; Lee et al., 2024). Sensitivity to boundedness arises spontaneously, even when it is irrelevant to task demands (Ji & Papafragou, 2022).

Dwell time measures offer a unique and powerful tool for bolstering evidence for the psychological reality of boundedness. If boundedness is a foundational property of event architecture, it follows that bounded and unbounded events should elicit systematically different dwell time patterns beyond a generalized “boundary advantage.” Furthermore, such shifts would occur not only during the segmentation of multiple sequential activities, but also *within* a single, segmented event. This is because bounded, but not unbounded events, possess a canonical beginning, middle, and end: in the event of folding a handkerchief, the event development follows the change of state of the affected object and the event ends when the handkerchief is fully folded; however, in the event of waving a handkerchief, the event has no discernible and predictable endpoint—and in principle, could go on forever. Even if both events are followed by another one (e.g., stretching), and hence a breakpoint ensues, the endpoint that characterizes what happened before that breakpoint cannot be reduced to a single notion: the last stages of folding correspond to canonical event completion, but the last stages of waving correspond to simple stopping. As a result, one would expect that the attentional advantage of event endpoints compared to other time points, such as middles or even beginnings, should be modulated by the boundedness of the event, with the boundary advantage being more pronounced (or even limited to) bounded events.

According to this line of reasoning, combining classic dwell time methods with an account of internal event temporality (or boundedness) could lead to a more highly articulated notion of “event structure” and “event boundary” beyond those used in the literature on event segmentation. Such an approach could also lead to differential treatment of varieties of “event endpoints” depending on the global ontological event representation they belong to. Finally, this integrated approach could show how computations of the temporal profile of an event impact online processes of event understanding.

### 1.3 Dwell times and signatures of event boundedness

The present study adopts the dwell time paradigm to directly test for signatures of boundedness in how attention is allocated during the moment‐to‐moment unfolding of an event. Unlike past dwell time studies that presented relatively long, hierarchically organized activity sequences (e.g., Hard et al., 2011: 156–247 slides; 2019: 2800 slides; Kosie & Baldwin, 2019a: 66–137 slides; 2019b: 57–112 slides; 2021: 20 slides), we focus on single events instantiated via very short image sequences. This departs from prior work that placed emphasis on attention to boundaries during event sequences to focus on what happens *within* the internal profile of events (particularly at final boundaries, i.e., endpoints). As mentioned already, we hypothesize that fine‐grained event structure lives within the edges of even such short exposures: in particular, bounded events—which have predictable and well‐defined internal structure culminating in an endpoint—should elicit more dramatic shifts in attention to endpoints compared to unbounded events—which lack such structure. In Experiment 1, we test two novel predictions that flow from this hypothesis. First, attention at the ends of events should be greater compared to beginnings for bounded events—but this difference should be less dramatic (or even entirely absent) for unbounded events. Second, attention at event ends should be greater compared to middles for bounded events—but again, this change in attention should be less pronounced (or even absent) for unbounded events. In Experiment 2, we ask whether the results replicate if participants had prior access to the content of the dwell time stimuli via a linguistic description (and whether the temporal structure of the description itself might affect their attention allocation).

## 2 Experiment 1

### 2.1 Method

#### 2.1.1 Participants

Forty‐three adult native speakers of English (19 female, 24 male, *M_Age_* = 43.2 years, *SD* = 13.4) were recruited via Prolific. The sample size was based on Hard et al. (2011). All participants in this and the next experiment provided informed consent. The experiment took about 4 min, and participants were compensated $0.55 for completing the study.

#### 2.1.2 Materials

Twelve pairs of videos between 6 and 9 s in length were adapted from Ji and Papafragou (2020a). Each pair included a closely matched bounded and an unbounded event with the same duration. Each video showcased the same actor performing an action on a tabletop against the same simple background (see Table 1 for a complete list). These videos belonged to a larger group of videos that had been normed by Ji and Papafragou (2020a, b) to confirm the contrast in boundedness between the paired events: (a) bounded events as a whole were considered to have “a beginning, middle, and ending” 87.2% of the time compared to 20.3% of the time for unbounded events (*t*(1, 39) = 20.05, *p* < .001); (b) bounded events prompted descriptions with change‐of‐state verbs (e.g., *fold a handkerchief*) or quantified count noun phrases (e.g., *tie a knot*) 98.2% of the time, while unbounded events prompted descriptions with verbs of activity (e.g., *wave a handkerchief*) or unquantified noun phrases (bare plurals or mass nouns: e.g., *tie knots*) 92.8% of the time. These videos were also normed to be equal in terms of intentionality, speed of the action, and visual similarity within pairs of bounded and unbounded events (ibid.; cf. also Vurgun, Ji, & Papafragou, 2024).^1^

**Table 1:** List of event stimuli

	Bounded events	Unbounded events	Duration (s)
1	cut a piece of paper in half	cut ribbon from a roll	6.40
2	roll up a towel	twist a towel	7.50
3	fill a glass with milk	shake a bottle of milk	8.27
4	crack an egg	beat an egg	6.00
5	fold a handkerchief	wave a handkerchief	8.00
6	tie a knot	tie knots	7.00
7	draw a balloon	draw circles	8.00
8	put up one's hair	scratch one's hair	8.00
9	tear a paper towel	tear slices off paper towels	8.00
10	stack a deck of cards	shuffle a deck of cards	6.33
11	blow a balloon	blow bubbles	9.00
12	group pawns based on color	mix pawns of two colors	7.50

**Fig. 1:**
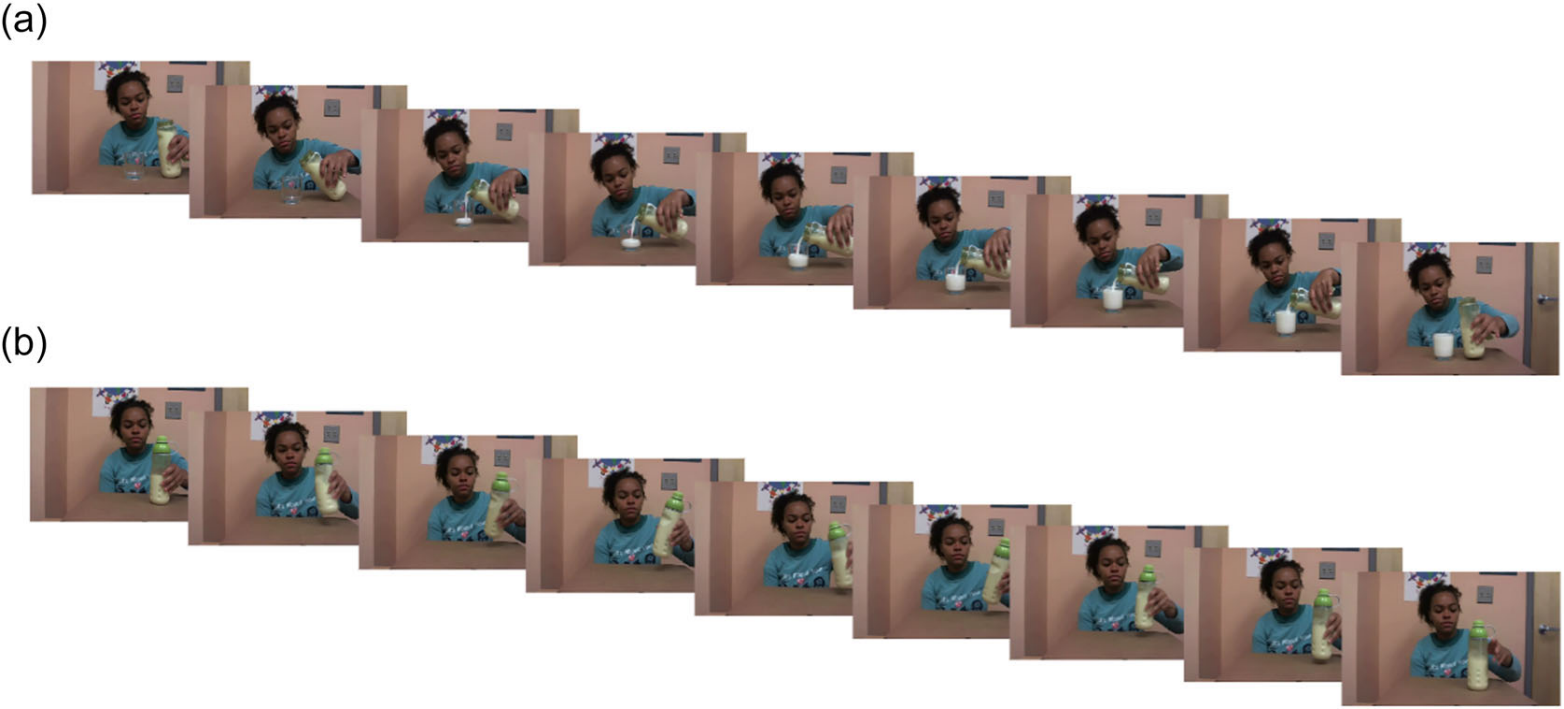
Sample dwell time trials: slideshow for a bounded event (fill a glass with milk; Panel a) and an unbounded event (shake a bottle of milk; Panel b).

Dwell time slideshows were created by selecting nine still images from each video within this set (see Fig. 1 for an example). The very first and last frames were extracted; seven additional still images were extracted at equal intervals between the first and last frames (at the rate of 10 frames per second). Because the video pairs varied in length, these seven images were sampled at slightly different rates across pairs (*M* = 1.22 fps; range: 0.98−1.91 fps). Overall, though, these sampling rates were similar to those in previous dwell time studies (Hard et al., 2011: 1 fps, Kosie & Baldwin, 2019a: 1 fps; Kosie & Baldwin, 2019b: 1 and 2 fps).^2^ The total set of nine images per slideshow chosen by our proportion method sufficiently and comprehensibly showcased the unfolding of each event.

The 12 pairs of slideshows were divided into two presentation lists. Each list contained only one member from a bounded‐unbounded event pair and had the same total number of bounded and unbounded events (six each), arranged in a pseudo‐randomized order. For both lists, we also created the same two practice slideshow trials with additional events, one unbounded (stir yogurt) and the other bounded (stick a sticker; both also drawn from Ji & Papafragou, 2020a).

#### 2.1.3 Procedure

The experiment was hosted on an online experiment‐building platform, PennController IBEX (Zehr & Schwarz, 2018). Participants received the following instructions: “In this experiment, you will see 12 videos. You will advance through the videos at your own pace by pressing the spacebar. You can spend as much time looking at each screen as you wish. Please pay careful attention to each video because you will be asked questions throughout the study about what you saw.” Participants were randomly assigned to one of the two presentation lists for the dwell time task. Dwell times were calculated per still image as the latency between the time point of image presentation and participants’ keypress to advance the display.

As a means to keep participants engaged, four memory trials were included throughout the experiment. Those appeared between 2 and 4 dwell time trials. In the memory trials, all participants were shown the same four images depicting the halfway point of a video: two of them were taken from the dwell time trials they had seen within the past 2–4 trials (avoiding the most recent ones within that set), and the remaining two images were new. These new images were taken from the other video within the bounded‐unbounded event pair participants had viewed during the trials (i.e., from the alternative presentation list; see also Table 1). In this way, participants across the two presentation lists for the main dwell time task could view the same four memory trials, but what would be new images for one group would be previously seen images for the other, and vice versa. Within each set of seen and new images, one showed a bounded and the other an unbounded event. The participants answered “Yes” if they thought they had seen the image and “No” if they did not. Prior to the main experiment, we presented participants with the same two practice dwell time trials and one practice memory question to familiarize them with the task.

### 2.2 Results

The data, analysis code, experimental code, and additional resources for this and subsequent experiments can be accessed at https://osf.io/rnsez. An initial analysis showed respectable accuracy on the memory trials (*M* = 77%). With this in mind, we turned to our main measure of interest. Prior to our main dwell time data analysis, dwell times faster than 80 ms and slower than 2500 ms were excluded from analysis. In accordance with procedures established by Hard et al. (2019, 2011) and Kosie and Baldwin (2019a, 2019b), dwell times greater than 3 standard deviations (SD) from the overall group mean were also excluded. Additionally, one participant garnered an overall mean dwell time greater than 3 SD away from the means of other participants and was excluded from the analysis. Overall, 6.5% of the original data was affected.

**Fig. 2:**
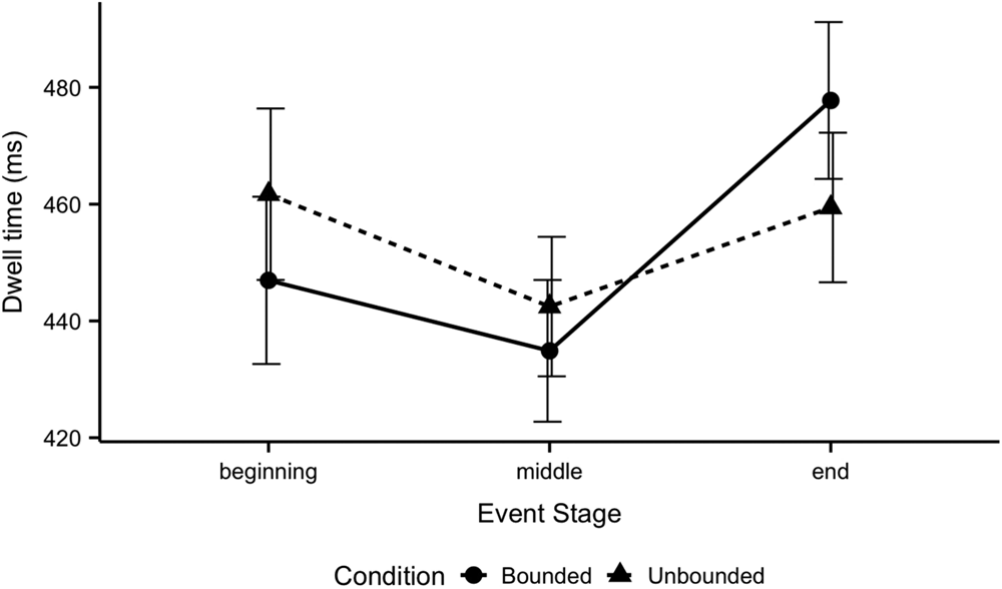
Average dwell times in Experiment 1 by Event Stage and Event Type. Error bars represent standard errors.

We divided the nine total slides evenly into thirds to represent three stages (beginning, middle, and end) within each event. Following past research (Hard et al., 2011; Kosie & Baldwin, 2019a, 2021), we then excluded data from the very first slide of each event because it always elicited very high dwell times compared to the rest (*M_bounded_* = 838 ms, *M_unbounded_* = 854 ms). This is known to occur because participants are adjusting to the task. Fig. 2 presents average dwell times at each event stage by event type for the remaining slides. We went on to analyze these data to test the two predictions at the core of our account. First, attention at the ends of events should increase compared to beginnings for bounded events—but this difference should be smaller or negligible for unbounded events. Second, attention at the ends of events should be greater than at the middles for bounded events—but again, this change in attention should be smaller or nonexistent for unbounded events.

To test our predictions, we analyzed dwell times from the beginning and middle versus the end of bounded versus unbounded events. We used linear mixed effects models with Event Type (contrast‐coded, Unbounded = 0.5, Bounded = −0.5), Event Stage (treatment coded with End set as the reference level, creating two contrast vectors [End = 0, Beginning = 1, Middle = 0], and [End = 0, Beginning = 0, Middle = 1]) and the Event Stage by Event Type interaction as fixed effects. As random effects, we entered intercepts for subjects and events, in addition to by‐subject and by‐event random slopes for the effects of Event Type, as well as Event Stage and their interaction, when justified by model comparison: Random effects began fully crossed and fully specified with by‐subject and by‐event slopes for relevant main effects. They were reduced (starting with by‐event effects) through model comparison such that only random effects that contributed significantly to the model (*p*<.05) were included (Baayen, Davidson, & Bates, 2008). Analyses were conducted using the *lme4* package (version 1.1.35.2) (Bates, Maechler, Bolker, & Walker, 2015) and *lmerTest* (version 3.1.3) (Kuznetsova, Brockhoff, & Christensen, 2017) in the R software environment (R Core Team, 2023). We used the same analytical method and approach for paired linear mixed effect model comparisons, where appropriate.

The statistical analysis is reported in Table 2. The analysis revealed a main effect of Event Type on overall dwell times: participants paid more attention to bounded events than unbounded events (*p* = .006). Additionally, the analysis identified a main effect of Event Stage on dwell times: participants paid significantly more attention to event ends compared to both event beginnings (*p* = .010) and middles (*p*<.001). Importantly, as we predicted, we found an interaction between Event Stage and Event Type for both contrasts of interest.

**Table 2:** Results of the lmer model for dwell times at the beginning versus ends, and middles versus ends of bounded versus unbounded events in Experiment 1

Effect	Estimate	SE	df	*t* value	*p* value
Intercept	472.82	46.89	43.01	10.08	<.001^***^
Event Type	−23.76	8.57	3927.91	−2.77	.006^**^
Event Stage (Beg‐End)	−17.39	6.77	3927.05	−2.57	.010^*^
Event Stage (Mid‐End)	−35.21	6.04	3927.03	−5.83	<.001^***^
Event Type:Beg‐End	41.91	13.55	3927.08	−3.09	.002^**^
Event Type:Mid‐End	30.47	12.08	3927.01	2.52	.012^*^

*Note*. Formula in R: Dwell Time ∼ Event Type * Event Stage + (1 | participant) + (1 | event).

* *p* < .05,

** *p* < .01,

*** *p* < .001.

To follow up the interaction, separate linear mixed effects models were performed with Event Stage as a fixed effect on data from bounded (Table 3) and unbounded events (Table 4). These analyses confirmed our predictions. Participants paid more attention to event ends compared to event beginnings for bounded events (*p* = .036) but no such difference emerged for unbounded events (*p* = .72). Similarly, participants paid more attention to event ends compared to event middles for both bounded (*p* = .005) and unbounded events (*p* = .014) but the difference was greater in the bounded cases (see Fig. 2).

**Table 3:** Results of the lmer model for dwell times during bounded events in Experiment 1

Effect	Estimate	SE	df	*t* value	*p* value
Intercept	486.22	52.61	46.36	9.24	<.001^***^
Event Stage (Beg‐End)	−39.80	17.78	22.13	−2.24	.036^*^
Event Stage (Mid‐End)	−52.00	15.42	12.81	−3.37	.005^**^

*Note*. Formula in R: Dwell Time ∼ Event Stage + (1 + Event Stage | participant) + (1 + Event Stage | event).

* *p* < .05,

** *p* < .01,

*** *p* < .001.

**Table 4:** Results of the lmer model for dwell times during unbounded events in Experiment 1

Effect	Estimate	SE	df	*t* value	*p* value
Intercept	461.17	47.32	44.76	9.75	<.001^***^
Event Stage (Beg‐End)	3.26	9.18	1938.19	0.36	.72
Event Stage (Mid‐End)	−20.06	8.17	1938.11	−2.46	.014^*^

*Note*. Formula in R: Dwell Time ∼ Event Stage + (1 | participant) + (1 | event).

* *p* < .05,

** *p* < .01,

*** *p* < .001.

### 2.3 Discussion

We examined how viewers’ dwell times changed as short, singular events unfolded over time to test two predictions about how event boundedness should affect online attention allocation. Both of our predictions were confirmed. First, viewers’ attention increased between beginnings and endings for bounded events, but no such difference emerged for unbounded events. Second, and relatedly, viewers’ attention was significantly higher at event endings compared to event middles, but this increase in attention was modulated by boundedness: the midpoint‐endpoint difference was more pronounced when viewing bounded than unbounded events. These results replicate earlier findings reporting an event boundary advantage in the dwell time literature (Hard et al., 2019, 2011; Kosie & Baldwin, 2019a, 2019b, 2021) but go beyond past work by showing that this advantage depends on the structure of distinct, abstract event types defined by the notion of boundedness.

## 3 Experiment 2

Many event segmentation studies have found remarkable consistency in segment length, organization, and even event boundary placement, regardless of observers’ prior knowledge—or even understanding—of the dynamic stimulus (Hard, Tversky, & Lang, 2006; Zacks et al., 2001). Other studies, however, have found that prior knowledge of an event (e.g., through local opportunities for repeat viewing) leads to reorganization of attention (Kosie & Baldwin, 2019a, 2019b). In Experiment 2, we replicated Experiment 1 but provided a sentence describing each bounded and unbounded event before participants saw them. We were interested in whether results from Experiment 1 would replicate if participants had access to such a linguistic preview.

We predicted that the inclusion of language in Experiment 2 would reduce the cognitive load of understanding the events during later visual apprehension, presumably because participants could better predict what would happen and spend less time inspecting each still image of the events. In other words, we expected linguistic previews to cause a decrease in overall dwell times in Experiment 2 compared to Experiment 1. Of primary interest was whether this decrease would depress or erase the boundedness‐driven differences in dwell times seen in Experiment 1. One possibility is that viewers would no longer consider event endpoints as information‐rich regions, especially for bounded events, since now the endpoint would be informed by the linguistic preview of the event. If so, the advantage of endpoints in bounded events might diminish or disappear in Experiment 2. Alternatively, previews may not affect the main patterns in Experiment 2 beyond speeding up viewing times.

A secondary question of interest was whether properties of the sentences used in previews might affect the data. We manipulated the grammatical aspect of the verb within each sentence (perfective vs. imperfective). Past work has shown that perfective and imperfective aspects can place emphasis on event cessation versus ongoingness, respectively (Madden & Zwaan, 2003). In our design, a cessation construal might enhance attention to endpoints, especially for bounded events.

### 3.1 Method

#### 3.1.1 Participants

For Experiment 2, 116 new adult native speakers of English (64 female, 51 male, 1 other, *M_Age_* = 36.9 years, *SD* = 12.7) were recruited via Prolific. Because this experiment used an additional manipulation, the sample size was larger than Experiment 1. The experiment lasted about 6 min, and participants were compensated $0.80 for completing the study.

#### 3.1.2 Materials

The same slideshows from Experiment 1 were used in Experiment 2. We additionally created English sentences describing each event in ways that corresponded to the descriptions of these stimuli in Table 1. For each sentence, we created two versions with different grammatical aspects (perfective vs. imperfective; e.g., *The girl filled/was filling a glass with milk* for the event in Fig. 1a; and *The girl shook/was shaking a bottle of milk* for the event in Fig. 1b). These versions were also used for a linguistic check, ensuring that participants processed the sentences (see below).

From each of the original two presentation lists of Experiment 1, we created two sublists of 12 videos each, depending on whether the event was described in the perfective or imperfective aspect prior to viewing. Each sublist crossed Event Type (bounded, unbounded) by Aspect (perfective, imperfective), both within subjects (with three trials for each Type × Aspect combination). Each sublist was arranged in a single, pseudo‐randomized order. Each participant was randomly assigned to one of four total presentation groups, depending on the sublist they saw.

#### 3.1.3 Procedure

Experiment 2 followed the same procedure as Experiment 1 with the addition of linguistic previews. At the beginning of each dwell time trial, participants saw a sentence for 6.5 s and were instructed to remember the exact sentence shown. After each dwell time trial, participants had to “choose the sentence they memorized” by selecting between the same sentence that they saw and the version of the sentence in the other grammatical aspect (i.e., perfective vs. imperfective aspect versions). This “linguistic check” ensured that participants would mentally entertain a verbal description of the event during incremental event viewing. Prior to the main experiment, participants saw two practice dwell time trials, two linguistic check trials, and one memory trial to familiarize themselves with the task.

### 3.2 Results

Memory performance was comparable to Experiment 1 (*M* = 78%). As for linguistic check accuracy, participants were, on average, 95% accurate in their memory of sentences. We next turn to dwell times as our main measure of interest.

As in Experiment 1, dwell times faster than 80 ms and slower than 2500 ms were excluded from analysis. Dwell times greater than 3 SD from the overall group mean were also removed. Furthermore, three participants garnered individual mean dwell times over 3 SD away from the individual means of other participants and were excluded. Five additional participants were excluded from analysis for not meeting accuracy criteria (<80% correct) for the linguistic check questions following each dwell time trial. Overall, 9.7% of the original data was affected. As in Experiment 1, dwell time data from the first slide were excluded from all analyses. We tested the same two predictions as in Experiment 1 (now also including Aspect as a factor in all our analyses).

#### 3.2.1 Results from Experiment 2

For our main analysis, we compared dwell times at the beginnings and middles versus ends of events using linear mixed effects models (lmer) with Event Type (contrast‐coded, Unbounded = 0.5, Bounded = −0.5), Event Stage (treatment coded with End set as the reference level, creating two contrast vectors [End = 0, Beginning = 1, Middle = 0], and [End = 0, Beginning = 0, Middle = 1]), Aspect (contrast‐coded, Perfective = 0.5, Imperfective = −0.5), as well as the Event Type by Aspect and Event Stage by Aspect interactions as fixed effects. The Event Type × Event Stage × Aspect interaction was also added as a fixed effect. As random effects, we entered intercepts for subjects and events, in addition to by‐subject and by‐event random slopes for the effects of Event Type, Event Stage, Aspect, and their interaction(s) when justified by model comparison: Random effects began fully crossed and fully specified with by‐subject and by‐event effects of Event Type, Aspect, Event Stage, and their interaction. Our approach to model selection was identical to Experiment 1.

The full statistical analysis is reported in Table 5. As in Experiment 1, we found a main effect of Event Type on overall dwell times, with bounded events overall attracting more attention than unbounded ones (*p* < .001). Additionally, the analysis identified a main effect of Event Stage on dwell times, such that event ends elicited more attention compared to event middles (*p*<.001) (but not compared to event beginnings, *p* = .45). Furthermore, as predicted, the analysis revealed a significant interaction between Event Type and Event Stage (*p*<.001 for both comparisons of interest). Aspect did not have an effect on dwell times, nor did it interact with other factors.

**Table 5:** Results of the lmer model for dwell times at the beginnings versus ends, and middles versus ends of bounded versus unbounded events in Experiment 2

Effect	Estimate	SE	df	*t* value	*p* value
Intercept	405.21	23.02	116.27	17.60	<.001^***^
Event Type	−20.64	4.80	10,152.02	−4.30	<.001^***^
Event Stage (Beg‐End)	2.86	3.79	10,151.01	0.76	.45
Event Stage (Mid‐End)	−34.07	3.39	10,151.01	−10.05	<.001^***^
Aspect	−1.27	4.80	10,151.70	−0.27	.791
Event Type:Beg‐End	26.67	7.59	10,151.02	3.52	<.001^***^
Event Type:Mid‐End	23.69	6.78	10,151.01	3.50	<.001^***^
Event Type:Aspect	−6.13	9.61	10,151.68	−0.64	.524
Beg‐End:Aspect	−3.53	7.59	10,151.01	−0.47	.642
Mid‐End:Aspect	0.21	6.78	10,151.01	0.031	.976
EvType:Beg‐End:Aspect	7.98	15.17	10,151.01	0.53	.599
EvType:Mid‐End:Aspect	11.82	13.55	10,151.00	0.87	.383

*Note*. Formula in R: Dwell Time ∼ Event Type * Event Stage * Aspect + (1 | participant) + (1 | event).

* *p* < .05,

** *p* < .01,

*** *p* < .001.

**Fig. 3:**
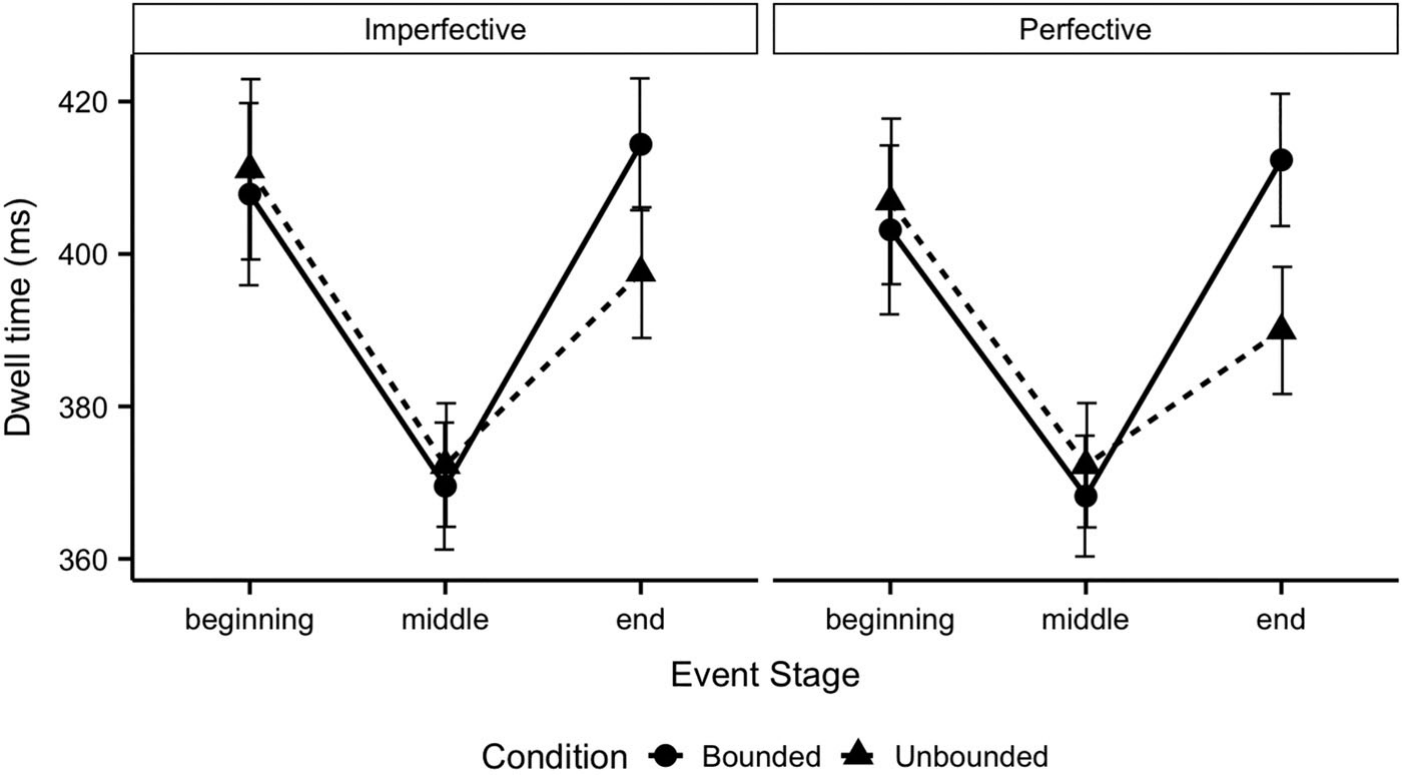
Average dwell times in Experiment 2 by Event Stage, Event Type, and Aspect. Error bars represent standard errors.

To pursue the interaction between Event Type and Event Stage, we built individual linear mixed effects models for bounded and unbounded events. The results are reported in Tables 6 and 7, respectively. For bounded events, there was a significant upward shift in attention from beginnings to ends (*p* = .049); however, for unbounded events, there was a significant *downward* shift in attention between beginnings and ends (*p* = .002; see Fig. 3). Furthermore, for both bounded and unbounded events, dwell time increased from middles to ends (both *p*s<.001), but the difference was much greater in bounded than unbounded events (see Fig. 3).

**Table 6:** Results of the lmer model for dwell times during bounded events in Experiment 2

Effect	Estimate	SE	df	*t* value	*p* value
Intercept	415.47	24.96	100.46	17.34	<.001^***^
Event Stage (Beg‐End)	−10.40	5.30	5025.10	−1.96	.049^*^
Event Stage (Mid‐End)	−45.93	4.74	5025.10	−9.70	<.001^**^

*Note*. Formula in R: Dwell Time ∼ Event Stage + (1 + participant) + (1 + event).

* *p* < .05,

** *p* < .01,

*** *p* < .001.

**Table 7:** Results of the lmer model for dwell times during unbounded events in Experiment 2

Effect	Estimate	SE	df	*t* value	*p* value
Intercept	395.13	23.88	89.27	16.55	<.001^***^
Event Stage (Beg‐End)	16.25	5.30	5016.11	3.06	.002^**^
Event Stage (Mid‐End)	−22.46	4.73	5016.10	−4.75	<.001^***^

*Note*. Formula in R: Dwell Time ∼ Event Stage + (1 | participant) + (1 | event).

* *p* < .05,

** *p* < .01,

*** *p* < .001.

#### 3.2.2 Comparison of results across experiments

**Fig. 4:**
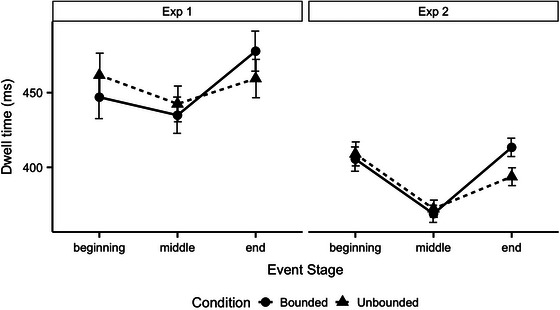
Average dwell times in Experiments 1 and 2 by Event Stage and Event Type. Error bars represent standard errors.

Recall that a linguistic preview was added in Experiment 2 to aid event apprehension. To see whether the addition had this effect, we compared dwell times across our two experiments (see Fig. 4 for a side‐by‐side presentation of the data). We used linear mixed effects models with Event Type (contrast‐coded, Unbounded = 0.5, Bounded = −0.5), Event Stage (treatment coded with two contrast vectors [End = 0, Beginning = 1, Middle = 0], and [End = 0, Beginning = 0, Middle = 1]), Experiment (contrast‐coded, Experiment 2 = 0.5, Experiment 1 = −0.5), and the Event Type by Experiment, Event Stage by Experiment, and Event Type by Event Stage by Experiment interactions as fixed effects. As random effects, we entered by‐event intercept and random slopes for the effects of Event Type, Event Stage, Experiment, and their interactions when justified by model comparison. The model output is reported in Table 8. We found that the inclusion of linguistic previews did influence overall dwell times: compared to Experiment 1, participants in Experiment 2 were overall much faster to look through each event (*p*<.001). There were no interactions with the Experiment.

**Table 8:** Results of the lmer model for dwell times at the beginnings versus ends, and middles versus ends of bounded versus unbounded events in Experiment 1 versus 2

Effect	Estimate	SE	df	*t* value	*p* value
Intercept	436.09	8.57	16.52	50.89	<.001^***^
Event Type	−19.13	8.80	14,243.56	−2.17	.030^*^
Event Stage (Beg‐End)	−5.08	6.95	14,243.04	5.13	.465
Event Stage (Mid‐End)	−31.41	6.20	14,243.03	−5.07	<.001^***^
Experiment	−65.02	8.79	14,243.02	−7.39	<.001^***^
Event Type:Beg‐End	28.52	13.90	14,243.07	2.05	.040^*^
Event Type:Mid‐End	24.57	12.40	14,243.02	1.98	.048^*^
Event Type:Experiment	−0.96	17.59	14,243.41	−0.054	.957
Beg‐End:Experiment	17.79	13.90	14,243.02	1.28	.201
Mid‐End:Experiment	−3.13	12.40	14,243.01	−0.25	.801
EvType:Beg‐End:Exp	−10.12	27.80	14,243.03	−0.36	.716
EvType:Mid‐End:Exp	−2.99	24.81	14,243.01	−0.12	.904

*Note*. Formula in R: Dwell Time ∼ Event Type * Event Stage * Experiment + (1 | event).

* *p* < .05,

** *p* < .01,

*** *p* < .001.

### 3.3 Discussion

Experiment 2 sought to uncover whether the attentional effects of Experiment 1 would replicate if participants had received a linguistic preview of the events they would see. As expected, the introduction of sentences describing to‐be‐viewed events in Experiment 2 resulted in faster dwell times than in Experiment 1, presumably because participants knew what would happen and spent less time inspecting each still image of the events. Nevertheless, Experiment 2 largely replicated the main findings from Experiment 1: event endings elicited more attention than event beginnings but only for bounded events. Furthermore, event endings elicited greater attention than event middles, but the increase in attention was larger for bounded compared to unbounded events. These patterns were unaffected by the specific grammatical form of the sentences that served as the preview of each event. In sum, even when top‐down event knowledge was available, and thus the event was globally more predictable, the internal, abstract type of each event (bounded vs. unbounded) robustly led to distinct online attention allocation patterns as viewers made sense of dynamically unfolding visual stimuli.

## 4 General discussion

### 4.1 Dwell times reveal signatures of event boundedness

We present evidence that the internal structure of events affects attention allocation during online event processing. Using a dwell time paradigm, we found that viewers allocated greater attention to event boundaries (ends) compared to nonboundaries (middles), replicating the boundary advantage that has been attested in the literature (Hard et al., 2019, 2011; Kosie & Baldwin, 2019a, 2019b, 2021). However, our data show that not all event boundaries are equal: viewers paid more attention as they neared the event end, resulting in longer dwell times compared to event middles, but this effect was more pronounced in bounded compared to unbounded events. The effect of boundedness also significantly interacted with viewers’ attention to the beginnings versus ends of events: event ends elicited greater attention than event beginnings, but only for bounded events. These effects on attention replicated across two experiments and persisted even when viewers had read linguistic descriptions (and thus had prior knowledge) of upcoming events.

These findings add to accumulating evidence that boundedness is a central feature of cognitive event architecture (Ji & Papafragou, 2020a, 2020b; Papafragou & Ji, 2024; see also Kuhn et al., 2021; Wellwood et al., 2018). Importantly, our findings also show that boundedness rapidly and systematically affects the online allocation of attention during an event. If we take the position that boundedness is fundamental to representing temporal entities (just like objecthood is fundamental for representing spatial entities; see Lee et al., 2024 for discussion), the fact that dwell times reveal signatures of boundedness indicates that an event's internal structure is recognized online during moment‐to‐moment event apprehension.

The present approach places the theoretical focus on the internal temporal texture *within* individual events as opposed to the breakpoints *between* events. As a result, it provides robust insights about the nature of any and every event, including how our representations of those events begin to evolve well before reaching a boundary, and how otherwise dissimilar event exemplars might have similar attentional signatures by virtue of falling under general event categories (such that, for instance, drawing a balloon, folding up a handkerchief, and filling a glass with milk have similar attentional profiles as members of the bounded class). Within these broad event classes, the present account leaves open the possibility that subtypes of events might emerge. Intuitions about boundedness can stem from the nature of the action itself. For example, folding a handkerchief leads to a well‐defined resultant state, but waving a handkerchief does not. The former is understood as a bounded event, and the latter unbounded event, despite the two events involving the same object. In some other cases, the source of boundedness can instead be found in the nature of the affected object. The event of tying a knot is a bounded event with a specific boundary imposed by the specific quantity of the affected object (one knot), whereas the event of tying knots does not have such a specific boundary.

Note that the present stimuli were created to be biased toward either a bounded or an unbounded construal. This was accomplished by either manipulating the type of action or the affected entity. Nevertheless, as mentioned already, (un)boundedness is a property of the human mind, not of stimuli in the world, and the same experience can often be construed from both a bounded and an unbounded perspective: an event where children are playing is unbounded, but an event where children are playing a game of chess is bounded. In a recent paper, linguistic framing of an event later influenced whether people interpreted a visual stimulus as bounded or unbounded (see Vurgun et al., 2024). It would be interesting to explore whether linguistic or other manipulations (e.g., aspectual features beyond those used in Experiment 2) might flexibly affect attention allocation to an event as measured by the dwell time paradigm. More broadly, it remains to be determined how the viewer's mind extracts boundedness categories from dynamic input, and how this process affects information‐processing at distinct time points within a single event.

### 4.2 Revisiting the “boundary advantage”

Why are endings of bounded events more privileged than endings of unbounded events? One possibility is that it is relatively easier to predict when a bounded event is going to end compared to unbounded events. Take, for example, a bounded event like filling a glass with milk. As long as the speed of the pouring remains consistent throughout the event, the fullness of the cup perfectly corresponds to the progression of the filling‐a‐glass‐with‐milk event. When the cup is 90% full, the event is typically 90% completed, and viewers can anticipate that the event is nearing its ending. Because viewers are better able to anticipate when they are approaching the endpoint, they can deploy increased attention for the upcoming event boundary.

Conversely, endings of unbounded events are less predictable. Take, for example, an unbounded event like shaking a bottle of milk. Perhaps we can, to a limited extent, predict when a shaking‐a‐bottle‐of‐milk event might end (from what we know about typical shaking‐a‐bottle events in the world). However, these predictions are less reliable. The shaking may only last for a very short time, or it may go on for longer than one would expect. Furthermore, the endpoint of this type of event might depend on the agent's (or another person's) unobservable goals or intentions, a factor that would make the predictability of such endpoints less well‐defined (on the role of intentionality on event boundaries, see Mathis & Papafragou, 2022). This means that in viewing unbounded events, it would be more difficult to anticipate when the event is going to end, and viewers would have decreased ability to efficiently increase their attention near the event boundary.

A deeper, related explanation for why endings of bounded events are more privileged than endings of unbounded events has to do with the content of the endings: the endings of bounded events contain important information for event understanding, since they correspond to a noteworthy change in the object involved in the event; by contrast, endings of unbounded events do not contain privileged information, at least in terms of content, because they do not involve meaningful changes. This line of reasoning is consistent with findings from an eye tracking study showing that, at the video offset, people pay more attention to the action and the object in resultative events that involve a high degree of (presumably predictable) change in an affected object compared to events with a less salient change of state (Sakarias & Flecken, 2019; cf. also Altmann & Ekves, 2019). Notice that, in our view, what counts as a change in an object over time can be abstract and depend on the viewers’ perspective: for instance, sometimes both bounded and unbounded events involve an outward change in an affected object (draw a balloon vs. draw doodles), but the boundedness construal will depend on whether the object is construed as an individual (e.g., a balloon) or a nonindividuated entity (doodles).

These specific patterns in the endpoint advantage are broadly compatible with the idea that predictability within an event determines attention allocation around boundaries (see EST; Zacks et al., 2007). Beyond this idea, however, the boundedness effect is not anticipated by current theories focusing broadly on the role of event boundaries in cognition (e.g., Zacks et al., 2007) but falls out from theories that recognize the role of the abstract internal structure of different ontological types of events in how events are processed and understood (e.g., Ji & Papafragou, 2023). Unlike classic notions of “event boundary” in event segmentation or dwell time studies that do not specify whether the event has reached its natural endpoint or has simply stopped, the notion of boundedness introduced here offers a more fine‐grained notion of boundary: even though both bounded and unbounded events can and do come to an end, the notion of “endpoint” represents something different in each case (reaching an inherent endpoint, i.e., culmination, as opposed to mere cessation, respectively). Similarly, the notion of beginning is different in each case, with beginnings in bounded events—but not unbounded events—being lawfully connected to a projected, structurally organized whole and eventual endpoint.

### 4.3 Expanding the dwell time paradigm in event cognition

Our experiment validates the use of dwell times as an implicit, rapid, and systematic index of online event processing. We now show for the first time that the dwell time paradigm can reveal not simply event structure in general but finely articulated subtypes of event ontology (and corresponding notions of what counts as an “event boundary”).

In the current study, systematic dwell time patterns emerged even with single isolated events that were considerably shorter and simpler than the activity sequences tested in earlier dwell time studies. In this sense, our findings indicate that viewers extract internal temporal event structure even for short, simple, already segmented everyday events, and this structure, in turn, rapidly affects the allocation of event‐internal attention. Future uses of the dwell time paradigm (e.g., with naturalistic stimuli obtained beyond the lab) have the potential to further illuminate how people process internal event structure and thus advance theories of event cognition. Beyond the current methods, we expect event boundedness to have further downstream cognitive consequences for how an event is mentally processed, remembered, and described. Finally, as shown by the comparison of Experiments 1 and 2, language can affect global dwell times for events: information about an event in a linguistically conveyed proposition can later help the processing of dynamic visual input. The relation between language and event attention is a fruitful direction for future work.

All raw data and the code for the analyses have been made available on the Open Science Framework (OSF) and can be accessed at https://osf.io/rnsez

## Notes

1 As the reader may notice, some of our unbounded events (tie knots) involve repetitions of a single, bounded event. Our argument is that such repetitions seem to continue indefinitely in our unbounded stimuli, and as a result, there is no discernible endpoint to the event, the structure is homogeneous. In bounded events, by contrast, the internal structure is differentiated, step‐wise, and hence nonhomogeneous (thus leading to a predictable endpoint). Even a well‐defined, bounded event (tie a knot), if placed in a chain of infinite repetition, will be construed as an unbounded entity (tie knots).

2 Hard, Recchia, and Tversky (2011) study selected the middle frame of 30 fps slideshows at each 1‐s intervals. However, the videos adapted for our study were significantly shorter in comparison and homed in on a singular event per trial (as opposed to several events in one sequence). We note that dwell‐time patterns, such as the boundary advantage, seem robust to slideshow resolution (Kosie & Baldwin, 2019b).
